# Supplementary figures and images for: Study on the effect of water content on physical properties of bentonite
Source: PLoS One. 2025 Jan 14;20(1):e0303522. doi: 10.1371/journal.pone.0303522 (PMC11731700; doi:10.1371/journal.pone.0303522)

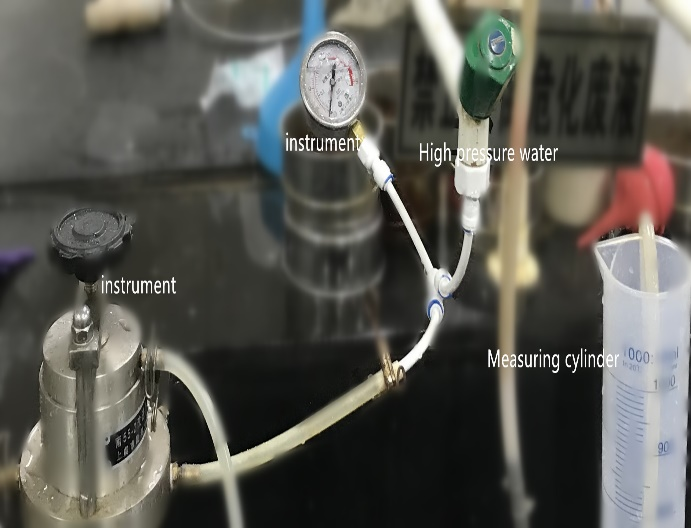

Supplement: S1 File — (ZIP) [file pone.0303522.s001.zip › 1a.png]

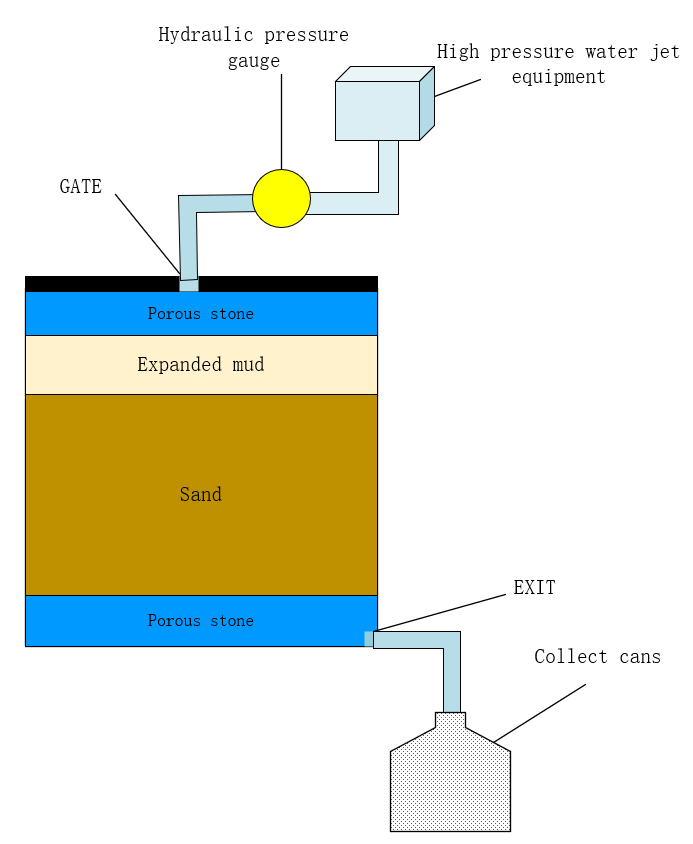

Supplement: S1 File — (ZIP) [file pone.0303522.s001.zip › 1b.png]

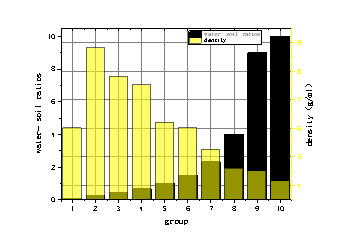

Supplement: S2 File — (PNG) [file pone.0303522.s002.png]

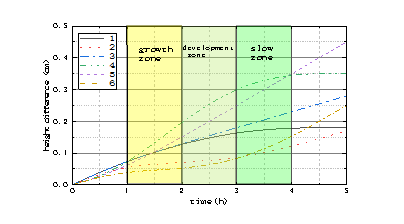

Supplement: S3 File — (PNG) [file pone.0303522.s003.png]

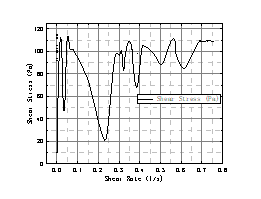

Supplement: S4 File — (ZIP) [file pone.0303522.s004.zip › 4a.png]

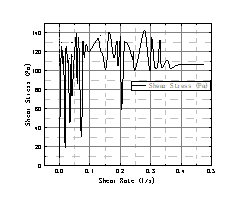

Supplement: S4 File — (ZIP) [file pone.0303522.s004.zip › 4b.png]

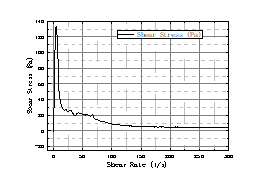

Supplement: S4 File — (ZIP) [file pone.0303522.s004.zip › 4c.png]

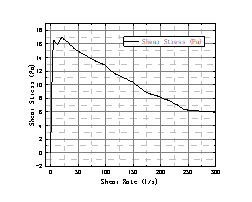

Supplement: S4 File — (ZIP) [file pone.0303522.s004.zip › 4d.png]

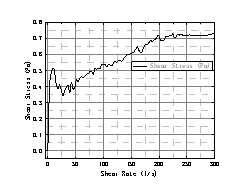

Supplement: S4 File — (ZIP) [file pone.0303522.s004.zip › 4e.png]

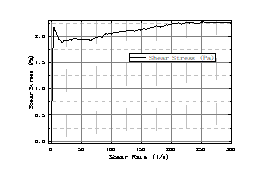

Supplement: S4 File — (ZIP) [file pone.0303522.s004.zip › 4f.png]

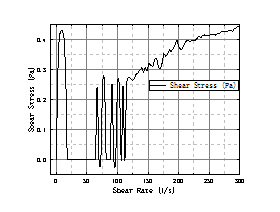

Supplement: S4 File — (ZIP) [file pone.0303522.s004.zip › 4h.png]

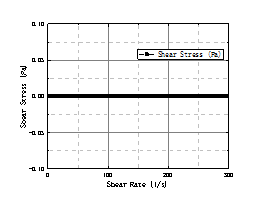

Supplement: S4 File — (ZIP) [file pone.0303522.s004.zip › 4i.png]

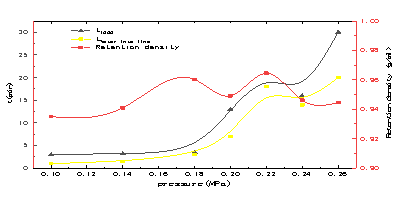

Supplement: S5 File — (PNG) [file pone.0303522.s005.png]

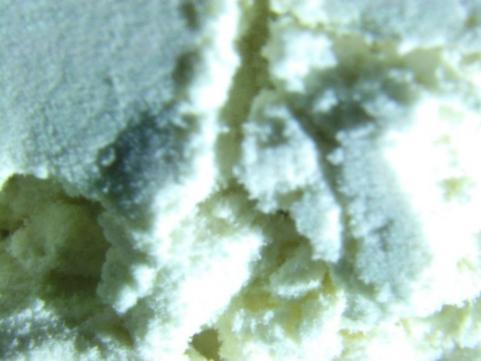

Supplement: S6 File — (ZIP) [file pone.0303522.s006.zip › 6a.png]

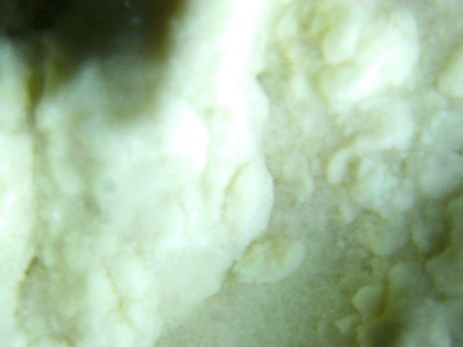

Supplement: S6 File — (ZIP) [file pone.0303522.s006.zip › 6b.png]

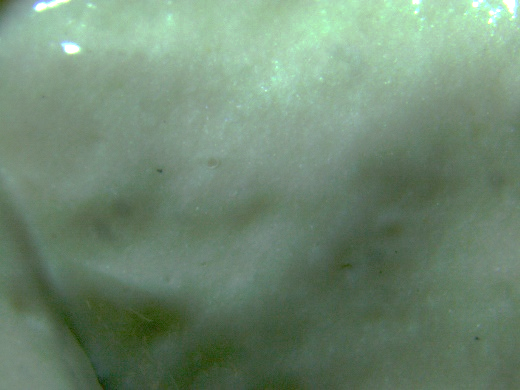

Supplement: S6 File — (ZIP) [file pone.0303522.s006.zip › 6c.png]

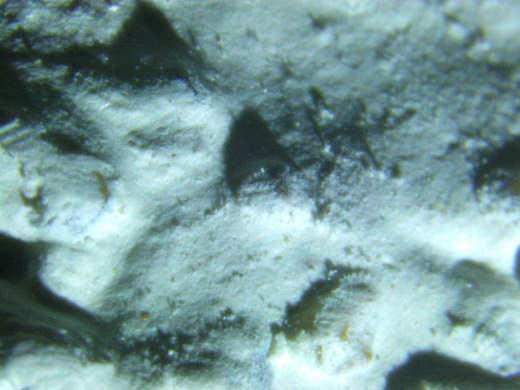

Supplement: S6 File — (ZIP) [file pone.0303522.s006.zip › 6d.png]

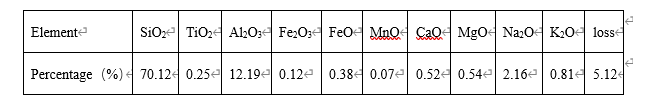

Supplement: S1 Table — (PNG) [file pone.0303522.s007.png]

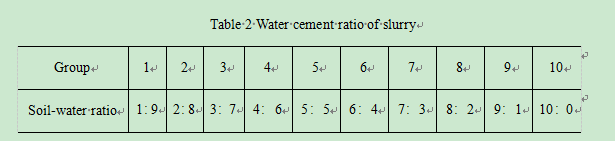

Supplement: S2 Table — (PNG) [file pone.0303522.s008.png]
