# Supplementary material for: Study on the effect of water content on physical properties of bentonite
Source: PLoS One. 2025 Jan 14;20(1):e0303522. doi: 10.1371/journal.pone.0303522 (PMC11731700; doi:10.1371/journal.pone.0303522)
Supplement: Attachment — Submitted filename: Reviewer Attachments for Manuscript Number PONE-D-24-00121.docx [file pone.0303522.s009.docx]

Based on the research results on the physical properties of bentonite in Nanyang, China, under different water contents, this paper will focus on the fundamental physical properties of bentonite and use a rheometer to measure the fluid properties of bentonite and use a stereomicroscope to comprehensively analyse the influence of water contents on physical properties of bentonite under different water contents. The overall content of the paper is relatively detailed, logical and fluent, and its research object, content and method are reasonable. But there are still the following problems:

1. Research background and literature review: The introduction section of the paper provides an overview of existing research, but lacks in-depth analysis of relevant research and existing literature. Suggest the author to expand the literature review section and discuss in detail the findings of previous studies and how they are relevant to current research.

2. When discussing the effects of different water contents on the physical properties of bentonite, the paper did not fully combine theoretical models or existing theories to explain the observed phenomena. For example, the discussion on how the rheological properties, permeability, and microstructure changes of bentonite are related to its chemical and physical properties at different water contents is not in-depth enough. It is recommended that the author refer more to and cite relevant theories when analyzing the results, in order to provide more in-depth and comprehensive theoretical explanations.

3. When describing the experimental process, the paper lacks detailed information about the experimental conditions and implementation steps, which can affect the repeatability and verifiability of the experimental results. For example, in the free expansion test and rheological property test, the preparation method of the sample, the environmental conditions of the experiment (such as temperature and humidity), and the specific steps of the experimental operation were not detailed. In order to improve the reliability and reproducibility of the research, the author should provide a more detailed description of the experimental methods.

4. The paper lacks sufficient statistical analysis to verify the significance and reliability of the data when presenting experimental results. For example, have repeated experiments been conducted to ensure consistency of results? Have statistical methods been used to analyze differences in data variability and significance? These are key elements to ensure the rigor of scientific research. The author should include relevant statistical analysis, such as mean, standard deviation, confidence interval, etc., when presenting the data to improve the credibility of the study.

5. Although the paper has studied the physical properties of bentonite at different water contents, there is a lack of sufficient comparative analysis with existing research. For example, the findings and conclusions in a paper should be compared with previous research to demonstrate how new research extends or challenges existing knowledge. Suggest the author to add a comparative analysis with existing literature in the discussion section, emphasizing the innovation and scientific contribution of this study。

6. The paper lacks sufficient discussion on the experimental scope and possible limitations. Any scientific research has its limitations, such as sample selection, limitations in experimental conditions, etc., which may affect the universality of results Performance and scope of application. In the paper, it seems that these potential limitations have not been fully identified and discussed. For example, factors such as the source of bentonite samples, particle size distribution, and variability in chemical composition may affect the experimental results. Suggest the author to discuss these limitations in detail in the discussion section and explain their potential impact on the research findings. Meanwhile, it is also possible to propose how to overcome these limitations in future work to enhance the broad applicability and depth of research.

Considering the above problems, it is recommended to the author make a major revision.
