# Supplementary material for: Study on the effect of water content on physical properties of bentonite
Source: PLoS One. 2025 Jan 14;20(1):e0303522. doi: 10.1371/journal.pone.0303522 (PMC11731700; doi:10.1371/journal.pone.0303522)
Supplement: Attachment — Submitted filename: Response to Reviewers.docx [file pone.0303522.s010.docx]

Response to Reviewers:

We would like to express our gratitude to the reviewers for their valuable feedback and constructive comments on our manuscript titled "Effects of Water Content on the Properties of Bentonite in Expansive Soils: A Case Study in the Longtan Temple Area, Chengdu."

To address the concerns raised by Reviewer #1:

1. Clarification of Investigated Properties: We appreciate the suggestion to specify the physical properties of bentonite studied under varying moisture conditions. In response, we have provided a detailed explanation of the properties investigated, including swelling potential, shrinkage, Atterberg limits, and permeability, in the revised manuscript.

2. Definition of "w saturated": Thank you for highlighting the need for a clear definition of the term "w saturated." We have added an explicit definition in the manuscript and explained how the saturation condition was achieved in our experiments.
3. Elaboration of Experimental Procedures: We acknowledge the need for a more detailed description of the experimental procedures. In the revised manuscript, we have provided a comprehensive account of the methods employed to control and measure water content, subject bentonite samples to various saturation levels, and assess changes in relevant physical properties.
4. Improved Summary of Findings: We have revised the conclusion to offer a clearer breakdown of the findings related to the saturated condition of bentonite. This includes explicit conclusions about the behavior of bentonite when saturated, thresholds identified, and the implications for mitigating hazards in expansive soil foundations and roadbeds.

To address the concerns raised by Reviewer #2:

1. Expanded Literature Review: We have expanded the literature review section to provide a more in-depth analysis of relevant research and discuss how previous studies are relevant to our research.
2. In-depth Theoretical Analysis: We have enhanced the discussion on the effects of different water contents on the physical properties of bentonite by incorporating relevant theoretical models and existing theories to explain observed phenomena more comprehensively.

Detailed Experimental Description: We have provided more detailed information about the experimental conditions and implementation steps to improve the repeatability and verifiability of our experimental results.

Statistical Analysis: We have included relevant statistical analysis, such as mean, standard deviation, and confidence interval, when presenting the data to improve the credibility of our study.

3. Comparative Analysis with Existing Literature: In the discussion section, we have added a comparative analysis with existing literature to highlight the innovation and scientific contribution of our study.

Discussion of Experimental Scope and Limitations: We have discussed the potential limitations of our study, including sample selection and experimental conditions, and proposed ways to overcome these limitations in future work to enhance the broad applicability and depth of our research.

We believe that these revisions address the concerns raised by the reviewers and significantly improve the quality and rigor of our manuscript. Thank you for the opportunity to revise and resubmit our work. We look forward to your feedback on the revised manuscript.

Sincerely,

Yuan Yanzhao
